# HVAC system attack detection dataset

**DOI:** 10.1016/j.dib.2021.107166

**Published:** 2021-05-28

**Authors:** Mariam Elnour, Nader Meskin, Khaled Khan, Raj Jain

**Affiliations:** a Department of Electrical Engineering, Qatar University, Qatar; b Department of Computer Science and Engineering, Qatar University, Qatar; c Department of Computer Science and Engineering, Washington University in St. Louis, USA

**Keywords:** Building management system (BMS), Smart building, Attack detection, Industrial control system (ICS), Cyber-physical system (CPS), HVAC system, Cybersecurity

## Abstract

The importance of the security of building management systems (BMSs) has increased given the advances in the technologies used. Since the Heating, Ventilation, and Air Conditioning (HVAC) system in buildings accounts for about 40% of the total energy consumption, threats targeting the HVAC system can be quite severe and costly. Given the limitations on accessing a real HVAC system for research purposes and the unavailability of public labeled datasets to investigate the cybersecurity of HVAC systems, this paper presents a dataset of a 12-zone HVAC system that was collected from a simulation model using the Transient System Simulation Tool (TRNSYS). It aims to promote and support the research in the field of cybersecurity of HVAC systems in smart buildings [1] by facilitating the validation of attack detection and mitigation strategies, benchmarking the performance of different data-driven algorithms, and studying the impact of attacks on the HVAC system.

**Specifications Table**

**Table:** 

Subject	Engineering
Specific subject area	Cybersecurity of industrial control systems and cyber-physical systems
Type of data	Table
How data were acquired	Simulation tool
Data format	Raw and analyzed data in three spreadsheet files
Parameters for data collection	hour of the year, hour of the day, temperature sensor measurements $T_{\text{zA}1}$ - $T_{\text{zA}4}$, $T_{\text{zB}1}$ - $T_{\text{zB}4}$, $T_{\text{zC}1}$ - $T_{\text{zC}4}$, $T_{\text{t}}$, $T_{\;\text{chiller}}$, $T_{\;\text{aoA}}$, $T_{\;\text{aoB}}$, $T_{\;\text{aoC}}$, $T_{\;\text{woA}}$, $T_{\;\text{woB}}$, $T_{\;\text{woC}}$, $T_{\text{amb}}$, control signals $U_{1}$ - $U_{13}$, setpoints, zones’ thermal comfort indices ${\text{PMV}}_{1}$ - ${\text{PMV}}_{12}$, total estimated power usage $P_{\text{total}}$, status of the HVAC system
Description of data collection	The data were collected from a simulation model of a 3-floor, 12-zone HVAC system using the Transient System Simulation Tool (TRNSYS), which is a user-friendly software that allows simulating the behavior of dynamic systems using energy and mass balance equations [2]. It has been widely used as a reliable tool for simulating the HVAC systems’ dynamics since its models were developed by authoritative departments to be consistent with practical data, and to reproduce the HVAC system to a large extent [3].
Data source location	Doha, Qatar
Data accessibility	Repository name: Mendeley Data Direct URL to data: https://data.mendeley.com/datasets/p63m3jrx9n/1
Related research article	M. Elnour, N. Meskin, K. Khan, and R. Jain, Application of data-driven attack detection framework for secure operation in smart buildings, Sustainable Cities and Society 69 (2021) 102816. doi: https://doi.org/10.1016/j.scs.2021.102816.

## Value of the Data

• The dataset is useful for anomaly detection and cybersecurity research for multi-zone HVAC systems in light of the increased threats on BMSs - which have the HVAC system as one of the major components-, the limited accessibility to real HVAC systems for research purposes, and the unavailability of public labeled datasets to investigate the cybersecurity of HVAC systems.
• It covers different models of attacks that can be launched against the HVAC system with different levels of severity.
• The dataset will be useful for promoting and supporting the study and research in the field of the security of intelligent buildings with respect to the most expensively operated equipment, namely the HVAC system.
• It can be used for benchmarking the performance of the various data-driven approaches for HVAC systems attack diagnosis and mitigation.
• It is useful to study the impact of the HVAC system malfunction on the efficiency of the system and the thermal comfort levels of the occupants.

## 1 Data Description

The dataset was collected from a simulated 12-zone HVAC system for cooling application. As presented in Table 1, it consists of three logs collected at a sampling rate of 1 min in which **Dataset log 1** contains normal operational data collected for four months - from June to September-, and **Dataset log 2** represents normal operational data collected for 20 days, and **Dataset log 3** consists of the normal and attack data of 16 attacks injected in a span of 20 days. The following variables were recorded: the hour of the year, the hour of the day, the measurements of 21 temperature sensors, 13 control signals, temperature setpoints, the 12 indices of the zones’ thermal comfort of occupants, the total estimated power used by the HVAC system, and the status of the system (i.e. 0 for normal operation and 1 for under attack). The detailed description of the variables is presented in Tables 2 and 3, and Table 4 shows the months in terms of the hour of the year. The attack models used were presented in [4], which are:

• **Attack 1:** Changing the setpoints of the control system
• **Attack 2:** Falsifying sensor measurements by freezing their values or introducing a bias
• **Attack 3:** Falsifying control signals by freezing their values or introducing a bias
• **Attack 4:** Modifying command signals to components

**Table 1:** Details of the HVAC system dataset.

Log	Type	Features	Number of samples
Data log 1	Normal	51 features: hour of the year, hour of the day, temperature sensor measurements, control signals, setpoints, system’s status.	194301
		
Data log 2	Normal	65 features: hour of the year, hour of the day, temperature sensor measurements, control signals, setpoints, zones’ thermal comfort indices, total estimated power usage, system’s status.	32161
		
Data log 3	Normal and attack	65 features: hour of the year, hour of the day, temperature sensor measurements, control signals, setpoints, zones’ thermal comfort indices, total estimated power usage, system’s status.	8840

**Table 2:** List of abbreviations used in the dataset.

Symbols		Subscripts	
T	temperature	z	zone
U	control signal	ao	output air
PMV	predicted mean vote	wo	output water
P	power	amb	ambient
t	time	y	year
		d	day

**Table 3:** The description of the data parameters.

Index	Symbol	Description
1	$t_{y}$	Hour of the year
2	$t_{d}$	Hour of the day
3	$T_{\text{amb}}$	The ambient temperature (${}^{\text{°}}C$)
4–15	$T_{\text{zA}1}$ - $T_{\text{zA}4}$, $T_{\text{zB}1}$ - $T_{\text{zB}4}$, $T_{\text{zC}1}$ - $T_{\text{zC}4}$	The temperature of the zones (${}^{\text{°}}C$)
16–18	$T_{\text{aoA}}$, $T_{\text{aoB}}$, $T_{\text{aoC}}$	The temperature of Air Handling Unit (AHU) supply air (${}^{\text{°}}C$)
19–21	$T_{\text{woA}}$, $T_{\text{woB}}$, $T_{\text{woC}}$	The temperature of cooling coil return water (${}^{\text{°}}C$)
22	$T_{\text{t}}$	The temperature of chilled water tank (${}^{\text{°}}C$)
23	$T_{\text{chiller}}$	The temperature of chiller outlet water (${}^{\text{°}}C$)
24–36	$U_{1}$ - $U_{13}$	The control signals
37–51	-	The temperature setpoints (${}^{\text{°}}C$)
52–63	${\text{PMV}}_{1}$ - ${\text{PMV}}_{12}$	The zones’ thermal comfort indices
64	$P_{\text{total}}$	The overall estimated power utilization of the HVAC system (kJ/h)
65	label	The label of the system status

**Table 4:** The months in TRNSYS in terms of the hour of the year.

Month	Start hour of the year	End hour of the year
June	3624	4344
July	4344	5088
August	5088	5832
September	5832	6552

The details of the attacks are presented in Table 5.

**Table 5:** Dataset log 3: List of injected attacks.

Attack index	Description	Attack time
1.1	Changing the setpoint of the chiller to 14 ${}^{\text{°}}C$	Day 1, 12:00
1.2	Changing the setpoint of the water tank to 16 ${}^{\text{°}}C$	Day 2, 06:00
1.3	Changing the setpoint of the AHU to 20 ${}^{\text{°}}C$	Day 2, 10:00
1.4	Changing the setpoint of Zone A1 to 26 ${}^{\text{°}}C$	Day 20, 11:00
1.5	Changing the setpoint of Zone C4 to 18 ${}^{\text{°}}C$	Day 1, 03:00

2.1	Freezing Zone B1 reading	Day 5, 16:00
2.2	Freezing Zone C4 reading	Day 7, 06:00
2.3	Freezing Zone A2 reading	Day 9, 04:00
2.4	Freezing Zone C3 reading	Day 10, 06:00
2.5	Introducing a bias of 3 ${}^{\text{°}}C$ to Zone B3	Day 3, 06:00

3.1	Freezing the control signal of Zone C2	Day 10, 15:00
3.2	Freezing the control signal of Zone B3	Day 13, 18:00
3.4	Freezing the control signal of Zone B1	Day 15, 06:00
3.5	Setting control signal of Zone B2 to 0	Day 19, 14:00
3.6	Setting control signal of Zone A3 to 1	Day 19, 20:00

4.1	Reducing the AHU-B water pump to 1/3 of its speed	Day 18, 12:00

## 2 Experimental Design, Materials and Methods

**Fig. 1:**
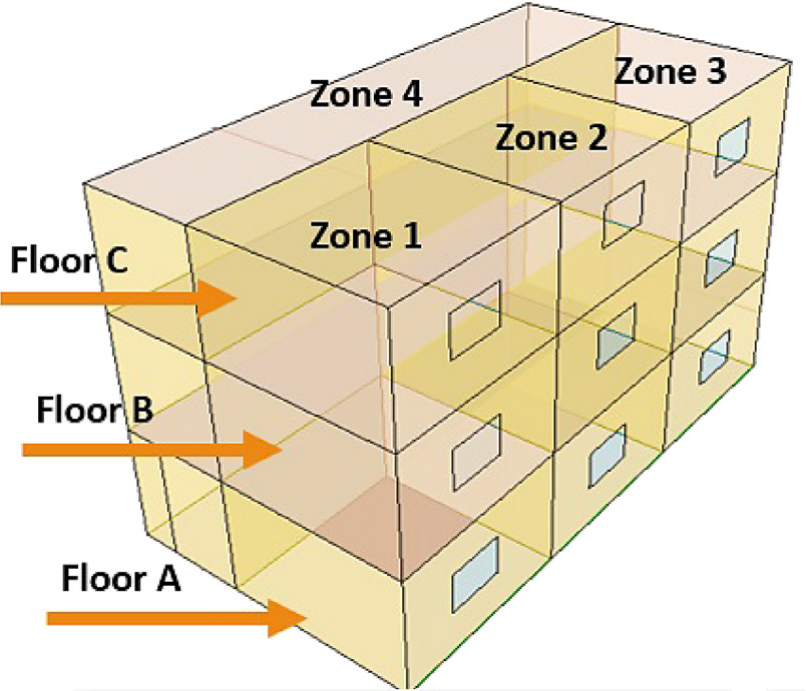
A sketch of the simulated 12-zone building [1].

**Fig. 2:**
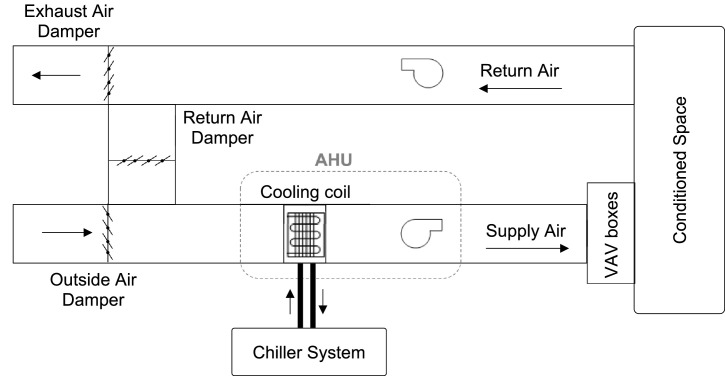
The diagram of a typical HVAC system using the Variable Air Volume (VAV) system [5].

As presented in [1], the building is a 3-floor office building operating from 6 AM to 6 PM. The floors are labeled A, B, and C and each floor consists of four zones where Zones 1-3 are office rooms and Zone 4 is a hall as shown in Fig. 1. It has a simple HVAC system for the cooling application as shown in Fig. 2 in which the temperature at each zone is controlled by proportional integral derivative (PID) controllers [5]. Each floor is equipped with an air handling unit (AHU) that provides the zones with cold air at a constant temperature of 13 ${}^{\text{°}}C$, and a variable flow rate controlled by the variable air volume (VAV) terminals. The chiller system and the cooling coils of AHUs are connected by the water tank that supplies chilled water to the cooling coils using a flow pump. The temperature of the chiller supply water $T_{\;\text{chiller}}$ is 9 ${}^{\text{°}}C$. The water tank temperature $T_{\text{t}}$ is controlled using a PID controller at 11 ${}^{\text{°}}C$ via a water valve to modulate the chilled water flow from the chiller to the tank.

It is challenging to obtain actual data or gain access to real building management systems due to confidentiality, unfeasibility, etc. Therefore, the use of reliable simulation tools is common and convenient to provide flexible means to conduct the research and analysis with high fidelity. Using the TRNSYS HVAC system simulation model, attacks were simulated by modifying the setpoint, sensor reading, or the control signal. HVAC systems are used to condition the indoor environment for occupants at minimum energy utilization. The HVAC system energy usage can be estimated by the consumption amount of the equipment such as the chiller, fans, and pumps. In terms of the thermal comfort level defined as the degree of satisfaction of occupants with the indoor thermal environment, the predicted mean vote (PMV) index is used to predict the mean response of a larger group of people according to the ASHRAE thermal sense scale [6] as presented in Table 6.

**Table 6:** The ranges of the PMV value for thermal comfort conditions.

Thermal sense	Hot	Warm	Slightly warm	Neutral	Slightly cool	Cool	Cold
PMV	+3	+2	+1	0	−1	−2	−3

The dataset can be used to facilitate validating attack detection and mitigation strategies, benchmarking the performance of different algorithms, and studying the impact of attacks on the HVAC system. As described in Table 7, four code files are provided as supplementary materials for training machine learning-based detection models using the Isolation Forest algorithm [1].

**Table 7:** The details of the supplementary code files.

index	File name	Description
1	HVAC - IF Training.ipynb	Developing an attack detection model using Isolation Forest on the raw data
2	HVAC - PCA-IF Training.ipynb	Developing an attack detection model using Isolation Forest on the data features extracted using Principal Component Analysis (PCA)
3	HVAC - 1D CNN Training.ipynb	Developing a feature extraction model using 1D Convolutional Neural Network (1D CNN)
4	HVAC - 1D CNN-IF Training.ipynb	Developing an attack detection model using Isolation Forest on the data features extracted using the 1D CNN model

## CRediT Author Statement

**Mariam Elnour:** Methodology, Software, Investigation; **Nader Meskin:** Conceptualization, Supervision, Writing - review & editing; **Khaled Khan:** Supervision, Writing - review & editing; **Raj Jain:** Writing - review & editing.

## Declaration of competing interest

The authors declare that they have no known competing financial interests or personal relation-ships that could have appeared to influence the work reported in this paper.
